# Relative Sensitivity and Quantitation in Glow Discharge Mass Spectrometry: A Progress Report

**DOI:** 10.6028/jres.093.089

**Published:** 1988-06-01

**Authors:** J. C. Huneke

**Affiliations:** Charles Evans & Associates, 301 Chesapeake Drive, Redwood City, CA 94063

Recent availability of commercial glow discharge mass spectrometer (GDMS) instrumentation, and the increasing industrial use of GDMS for bulk trace element characterization have necessitated rapid progress in understanding the systematics and requirements of GDMS analysis. GDMS approaches the ideal instrument for broad spectrum trace element analysis to ppbw levels. In the GDMS source the sample is the cathode for a dc discharge supported by 1 Torr of Ar or other gas. The composition of the atoms sputtered from the sample surface is the same as the bulk solid, providing a representative pool of atomized material for further analysis. Atoms are ionized in the plasma mainly by collision with metastable Ar atoms with energy levels near 11.5 eV, which is sufficient to ionize all but a few elements. Sputtered atoms diffuse through the plasma to the walls of the chambers, and ions formed near the exit orifice and leaving the chamber are mass analyzed and the mass separated ion currents measured. Ion currents out of the source are stable and high, and a few ion counts of an element can correspond to concentrations of less than a ppbw. For example, it has been determined that Fe present in Cu at a level of 5 ppbw can easily be measured to a precision of 10%.

With a few exceptions, GDMS ion yields vary by only an order of magnitude over the whole periodic table. Simple elemental survey analyses of solids can be provided by GDMS which are accurate to within the order of magnitude variation of relative GDMS yields. However, if material must be qualified within specified impurity limits or more accurate elemental contents are required, better measurement becomes essential and accurate relative sensitivity factors must be determined. There are no adequate theoretical or semi-empirical models of ion production out of the GDMS source yet available. Nor is there a good understanding of the effects of variations of physical factors on ion yield (i.e., pressure, discharge voltage and current, sample cell geometry, plasma gas composition). Relative elemental sensitivities must be determined by analysis under similar conditions of a standard material.

There are several constraints on appropriate standard material for the relative sensitivity determinations: (1) The full element survey capability of GDMS requires the widest possible element coverage for a specific matrix type, preferably within a single sample. (2) The ability to measure precisely elements present to the 10 ppbw level, coupled with the fact of analytical back contamination of 1E-3 to 1E-6 of the previous sample, requires accurate standard concentrations of 1–10 ppmw or less. (3) To ensure representative sampling suitable dopants are those dissolved in the matrix and not exsolved to grain boundaries or incorporated in separate phases since elements present in different phases may be sampled at different rates. (4) The physical shape of the standard and analytical samples must be similar to assure similar discharge conditions. (5) Standards must be characterized for sampling on the scale of 10–50 mm^2^ surface area at a rate of 1 μm/min. Since commercial GDMS is recent, standards generated for other methods must be used. Typically, available standards useful for GDMS characterization are designed (1) for analysis of larger areas, (2) for techniques less susceptible to heterogeneous distributions in multiphase assemblages, (3) are not particularly well characterized for elements below 10–100 ppmw concentration, and (4) are limited in the variety of dopant/matrix combinations.

The pattern of relative GDMS yields from an Ar plasma has been established using available standards, which are generally accurate to better than 10% for elements present at 10 ppmw or greater concentration. (Relative sensitivity factors as normally defined for quantitation purposes are the inverse of the relative GDMS yield illustrated in fig. 1.) There is a pronounced trend in yields across the group b transition elements, decreasing by an order of magnitude from high yields in groups 3b–5b to low yields in groups 1b–2b. The trend is general and not monotonic. A similar but separate trend is also observed for group a elements.

Relative GDMS yields determined using different standards of the same metal agree well within stated concentration errors, demonstrating an accuracy of GDMS analysis of 10–20%. Relative GDMS yields determined on the same metals but in a different laboratory (same manufacture of instrument) are generally quite comparable, but there are systematic differences by factors of two or three in the more electronegative elements, differences which are not well understood. Relative yields appear to be moderately sensitive to power levels and especially sensitive to plasma pressure. Until interlaboratory differences are resolved, relative GDMS yields determined on one instrument are accurate to no better than a factor of two for use on a comparable instrument.

The relative GDMS yields determined from a variety of standard metals (Al, Cu, Zn, Steel) typically agree within 30%, with Ni and Zn in Cu as notable exceptions indicating possible certification problems. The GDMS measurement is indeed matrix independent, as well as presently can be determined, and relative GDMS yields for application to one type of matrix can be reliably determined from a different standard type. The differences in the relative yield determinations from different metals may be due in no small part to the segregation of many of the elements into separate phases (clearly observed by SIMS imaging) with the consequence that sampling is not entirely uniform. Variations in GDMS measurements due to such segregation can be clearly exhibited and can be significantly reduced by sampling larger areas and by multiple sampling in a single measurement.

Regardless, not all elements are represented in standard metals, and theory or a suitable semiempirical model must be invoked to obtain the relative GDMS yields necessary for quantitation. In a promising approach it has been demonstrated that a Saha-Eggert formalism exhibits the general features of the GDMS yield pattern (W. Vieth, pers. comm.). Relative GDMS yields calculated this way are improved compared to uniform yields, particularly for group b elements, but again accuracy is no better than a factor of three for the more electronegative elements.

In summary, while the precision of GDMS measurements is excellent for the measurement of almost all elements at concentrations as low as a few ppbw, the accuracy of trace constitutent analysis is quite variable (10–300%) depending on the source of the relative GDMS yields needed for quantitation. The instrument itself appears inherently more capable than can be demonstrated using presently available standards. Improved assessment of GDMS performance and precise relative GDMS yield determinations will require more careful selection and characterization of standard materials.

## Figures and Tables

**Figure 1:**
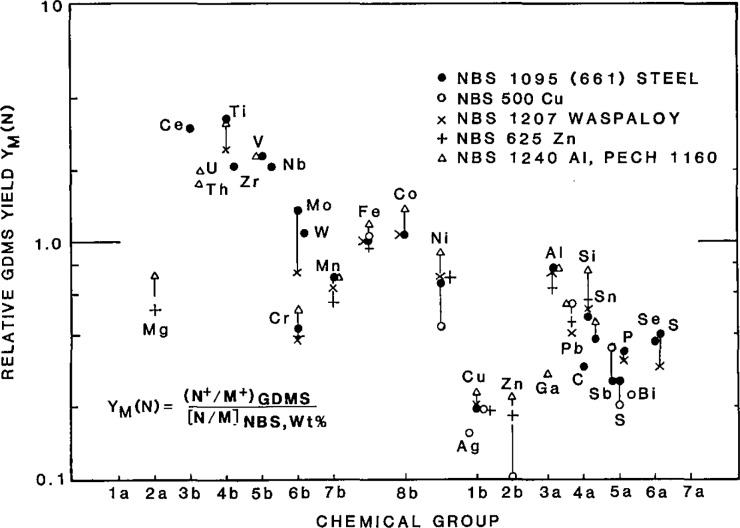
Relative GDMS yield from the glow discharge mass spectrometer analysis of several standard materials. Measurements were done at the same glow discharge power and pressure for each sample. Measured element ion currents are normalized to the *weight* fraction of the element in the standard to determine relative yield.

